# A Deformation Reconstruction Strategy for Integrated Truss Structures Subjected to Thermal–Mechanical Load

**DOI:** 10.3390/s25020558

**Published:** 2025-01-19

**Authors:** Zexing Yu, Xiaofei Ma, Jialong Zhu, Dayu Zhang, Yonggang Xue, Pengfei Huang, Yichen Li, Hao Li

**Affiliations:** Xi’an Institute of Space Radio Technology, Xi’an 710100, China; yuzexing@mail.nwpu.edu.cn (Z.Y.); zhujl_cast@163.com (J.Z.); 18092166275@163.com (Y.X.); pengf_huang@outlook.com (P.H.); yichenli10@163.com (Y.L.); lh651628333@stu.xjtu.edu.cn (H.L.)

**Keywords:** deformation reconstruction, large space truss, thermal–mechanical load, Ko displacement theory, structural health monitoring

## Abstract

The deformation monitoring of integrated truss structures (ITSs) is essential for ensuring the reliable performance of mounted equipment in complex space environments. Reconstruction methods based on local strain information have been proven effective, yet the identification faces significant challenges due to variable thermal–mechanical loads, interactions among structural components, and special boundary conditions. This paper proposes a deformation reconstruction strategy tailored for ITSs under combined thermal–mechanical load scenarios wherein deformations of both the primary truss structures and the attached panel systems are investigated. The proposed approach utilizes Ko displacement theory as the core algorithm, while the least squares optimization method is employed to determine the integration with unknown initial values during the reconstruction process. Validation is conducted through diverse load scenarios, and the reconstruction results are evaluated using errors based on the root mean square. The result demonstrates that the proposed method can reconstruct deformations of truss structures under both mechanical and thermal loads. Furthermore, the optimization-based approach achieves accurate reconstructed results in the case of panels with two-point fixed boundary conditions. This study provides an effective strategy for in-orbit deformation reconstruction, addressing challenges posed by complex loads and structural configurations.

## 1. Introduction

Large space truss structures play a critical role in large scale spacecraft by serving as a foundation, extension, support, and form maintenance system because of their excellent stowage and deployment performance, lightweight design, and high stability [1]. The structure can provide attachment points for CubeSats, solar arrays and other external payloads, in which the assembly is commonly referred to as integrated truss structures (ITSs) [2,3,4]. During in-orbit service, ITSs are in a complex space environment and face extreme working scenarios such as high vacuum and cyclic thermal loads [5]. The condition results in a non-negligible deformation of the structure and can significantly impair the craft’s performance, including the pointing accuracy of antennas, the imaging quality of telescopes, etc. For instance, the Hubble Space Telescope requires pointing stability of less than 0.007 arcsec during its operation [6], which imposes high demands on the measurement and control of deformation in the supporting truss structure. In addition, deformation may cause fatigue damage, ultimately shortening the structural lifetime. Hence, in-orbit monitoring of the deformation of ITSs is crucial for ensuring performance [7,8,9].

The deformation of ITSs is generally reconstructed based on the strain information since the deformation is difficult to measure directly during service [10]. With the rapid advancement of Fiber Bragg Grating (FBG) sensor technology, it has become feasible to achieve cost-effective, multi-point in-orbit monitoring over long distances [11,12]. However, the measured strain data are discrete and provide only local inferences about the structure. To tackle this issue, various algorithms have been developed to reconstruct the overall deformation based on local strain measurements [13,14,15]. One of the well-developed algorithms is the modal transformation method, which is based on the theory of superposition of structural mode shapes [16,17,18,19]. In the method, strain mode shapes and displacement mode shapes are combined to realize the conversion from the structural surface strain to the displacement. This strategy can reconstruct the deformation of all desired degrees of freedom (DOFs) simultaneously. However, the accuracy of the method is highly dependent on the numerical model, although some model correction studies have been carried out to minimize the discrepancy between the numerical model and the realistic structure. Moreover, the discrepancy between the moduli spaces of the displacement and strain fields threatens the accuracy of the deformation reconstruction. Local modalities and nonlinearity also limit the application of the strategy [10]. The second method is the inverse finite element method (iFEM), which was first proposed by Tessler and Spangler in 2003 [20,21]. The method employs a least squares optimization to minimize errors between numerical and measured strain components. Several iFEMs are carried out to reconstruct deformations of beams, plates, and frame structures [22,23]. Abdollahzadeh et al. proposed a quadrilateral inverse shell element to reconstruct the deformation of composite laminates in the case of geometrically nonlinearity [24], in which post-buckling and large deformation scenarios are considered. In addition, pre-extrapolation was used in their study to enlarge the stain information. For beam elements of iFEM, theoretically, six different surface-measured strains at a section are necessary to calculate the element strain component at a position, which may be reduced to two or three depending on the specific load case. However, the configuration of strain sensors dominates the accuracy and stability of the iFEM, where angles of sensors may result in singularities or ill-conditioning. To address this problem, Zhao et al. proposed an optimal strategy based on the particle swarm optimization method which was validated in a wing-like framework [25]. Errors in the process of pasting the sensors still impair the efficiency of iFEM. Moreover, they proposed a nonlinear iFEM based on strain gradient theory to meet the requirement of large displacement of the geometric nonlinearity [10], which does not involve linearized processing and results in the improvement of the stability of the proposed method. The numerical and experimental studies are carried out in the case of the transverse shear load and bending moment, and the priority of the method is revealed. Recently, Zhao et al. developed equivalent layered composite beams based on the generalized layered global–local beam theory to tackle limitations of the classical iFEM applied to beams with complex cross-sections [26], where the interlaminar continuity of shear stresses and displacements is considered. The results indicate that the method has an accuracy improvement of 5–8% compared to the classical method. The iFEM has the potential to reconstruct the deformation of large complex structures without the requirement of information on loads or material properties [27,28]. For beam-like structures, however, iFEM requires a high density of sensors to compute the strain component at each measurement point. Additionally, thermal loads also induce difficulties in the derivation of shape functions of the iFEM, which makes it hard for the method to reconstruct the deformation in real time [29,30,31].

The reconstruction method based on Ko displacement theory is based on the classical Euler–Bernoulli beam theory [32]. An accuracy reconstruction can be carried out along a one-dimensional beam by integrating discrete strain with piecewise continuous polynomials [33]. Ding et al. developed a strain-deformation reconstruction method for composite laminates based on Ko displacement theory [34]. The proposed method has been validated through both numerical and experimental analyses, where both concentrated and uniform load scenarios have been studied. Results demonstrate excellent capabilities in deformation reconstruction. In addition, error source analysis was performed in their study. Xing et al. developed a deformation reconstruction method for beam structures under pre-deformation conditions [35]. In their study, FBG sensors were used to capture beam strains, and Ko displacement theory was extended to overhanging boundary conditions. Numerical analysis and experimental tests demonstrate the potential of Ko displacement theory for more complex boundary conditions. Algorithms based on Ko displacement theory can reconstruct structural deformations without prior information on material properties with excellent accuracy and efficiency, which is one of the promising methods for in situ deformation identification in orbit. However, this approach is currently applicable to laboratory boundary conditions such as cantilevers, fixed supports, or simple supports.

Moreover, flourishing data-driven methods also provide more insights into deformation reconstruction [36,37], which negates the standard hypothesis and classic theories. Ding et al. adopted the back propagation (BP) neural network to establish the nonlinear mapping relationship between the measured strain and deformation [38]. In their study, cantilever composite laminates with equal thickness and variable thickness are studied, respectively, in which the load is applied at the end by using an indenter. The FBG is implemented as the strain sensor in the study. Moreover, the transferability of the BP neural network is validated by different test pieces. Fu et al. combined the iFEM and the solidified fuzzy network to reconstruct the dynamic deformation of a variable section wing with FBG sensors [39]. In their study, errors induced by FBGs and the dynamic unmodeled error are corrected by a linear support vector regression fuzzy network. Numerical and experimental results demonstrate the efficiency of the proposed algorithm. However, the versatility of the method needs to be further improved. Until now, the lack of a reliable in-orbit database has led to difficulties in training data-driven methods.

In the case of ITSs considered in this study, the complex structure brings some challenges to the deformation reconstruction. On one hand, non-symmetric designs in the geometries and material of space truss structures may result in bending–twisting coupling deformation. Simple curve-fitting methods may fail to accurately capture the deformation behavior of various components of the structure. In addition, accessories, such as antenna reflectors and optical instruments, mounted to the truss structure are deformed under the space environment, which acts as multi-point forces to the supported truss. On the other hand, to the best of the authors’ knowledge, published algorithms mainly study the deformation reconstruction of structures in experimental boundary conditions, such as cantilever and simple support. However, in real engineering applications, boundary conditions of ITSs and accessories are more complex. Consequently, the accuracy of reconstruction results based on these simplified boundary conditions is inadequate, as demonstrated in Section 2. Furthermore, the deformation of ITSs is mainly caused by cyclic thermal loads during service, whereas most existing studies primarily address simpler mechanical loads, such as multi-point or uniformly distributed forces. The strain distribution induced by thermal loads differs significantly from that caused by mechanical loads, and the effectiveness of current reconstruction methods under thermal conditions requires further validation.

To address these challenges, this study proposes a deformation reconstruction strategy for ITSs that considers both thermal and mechanical loads. The paper is structured as follows: Section 2 presents the theoretical framework of the proposed method, which is tailored to the specific characteristics of assembly structures. Section 3 details the numerical model and describes various load scenarios based on practical engineering applications. Section 4 validates the proposed deformation reconstruction method and results are discussed. Finally, conclusions are summarized in Section 5.

## 2. Deformation Reconstruction Method for ITSs

**Figure 1:**
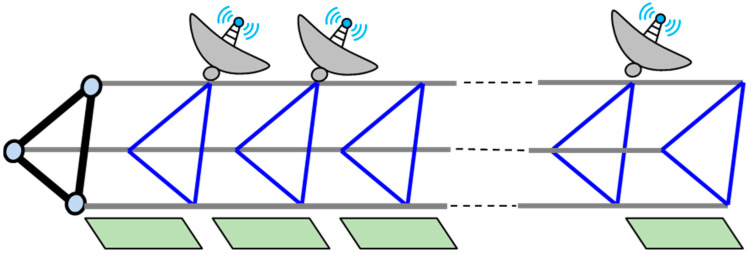
The diagram of integrated truss structures (ITSs).

The structure considered in this study is depicted in Figure 1. The space truss serves as the main foundation and support with its left end fixed to the spacecraft platform. Accessories such as CubeSats are mounted on one side of the truss structure, while plate-like structures, such as solar panels, are installed on the opposite edge. For ITSs, the straightness of the space truss and the flatness of the plate-like structure are critical indicators of in-orbit performance. Consequently, this study focuses on reconstructing the deformations of these two structural components, accounting for the interaction between accessories and other components as concentrated forces. For the truss, which is a typical beam structure, deformation may involve bending–twisting coupling due to its asymmetry. For the plate-like structure, its out-of-plane deformation can be represented as a combination of deflections from multiple one-dimensional beams. Theoretically, the reconstruction method based on Ko displacement theory shows potential for accurately identifying the deformation of ITSs under combined thermal and mechanical loads.

**Figure 2:**
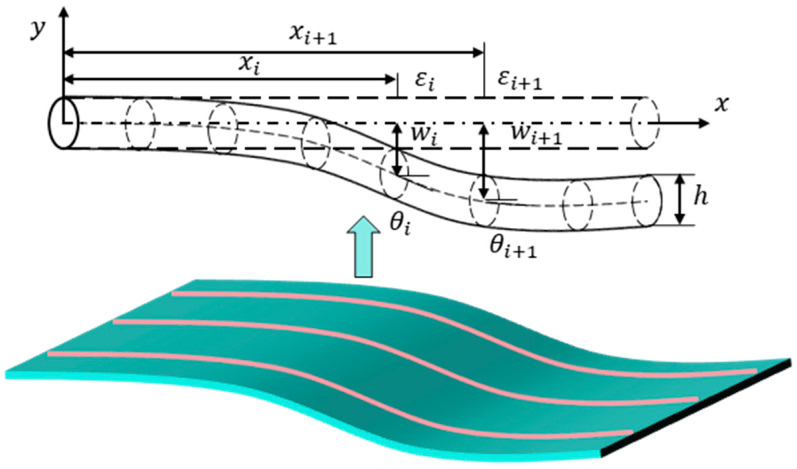
The schematic of Ko displacement theory.

The fundamental concept of Ko displacement theory is illustrated in Figure 2. Each curve is discretized into *n* beam segments, where the strain is assumed to be linearly distributed within each segment, as the segment length ΔL is sufficiently small. According to the mechanics of materials, the relationship between the deflection and strain in the local coordinate system can be expressed as follows:

(1) $$\frac{d^{2}w(x)}{dx^{2}}=\frac{\varepsilon (x)}{c}$$

where w(x) and ε(x) represent the deflection and strain at the coordinate x, respectively. c denotes the distance between measured surface and mid-surface, which is defined as

(2) $$c=\frac{\varepsilon_{t}}{\varepsilon_{t}-\varepsilon_{b}}h$$

where $\varepsilon_{t}$ and $\varepsilon_{b}$ are the measured strains at the top and bottom surfaces, respectively. h is the thickness. Generally, when bending dominates the structural deformation, the relationship between $\varepsilon_{t}$ and $\varepsilon_{b}$ is $\varepsilon_{b}=-{\;\varepsilon }_{t}$. Hence, the expression of c in Equation (2) can be simplified as

(3) c=h/2

Based on the linear hypothesis, strain ε(x) between two strain sensors $\varepsilon_{i}$ and $\varepsilon_{i+1}$ can be calculated by the measured strains:

(4) $$\varepsilon \left(x\right)=\varepsilon_{i}+(\varepsilon_{i+1}-\varepsilon_{i})\frac{x-x_{i}}{∆L}$$

The slope can be obtained by integrating Equation (4) in $x_{i}<x<x_{i+1}$:

(5) $$tan\theta_{x}=\frac{dw}{dx}=\int_{x_{i}}^{x_{i+1}}\frac{d^{2}w(x)}{dx^{2}}dx+{tan\theta }_{x_{i}}$$

where ${tan\theta }_{x_{i}}$ is the slope at the beginning position. According to Equation (5), the deflection between two sensors is expressed as

(6) $$w\left(x\right)=\int_{x_{i}}^{x_{i+1}}tan\theta \left(x\right)dx+w_{i}=\int_{x_{i}}^{x_{i+1}}\int_{x_{i}}^{x_{i+1}}\frac{\varepsilon (x)}{c}dxdx+\int_{x_{i}}^{x_{i+1}}{tan\theta }_{x_{i}}dx+w_{i}$$

where $w\left(x\right)$ is the displacement at the coordinates x. It can be seen that the deflection $w\left(x\right)$ is an integral function with variable limitation, which can be determined once values <$\;w_{i}$, ${tan\theta }_{x_{i}}$> at the lower boundary $x_{i}$ are known. The entire deformation of beams or panels can be reconstructed by sequentially splicing deflections of each beam segment.

**Figure 3:**
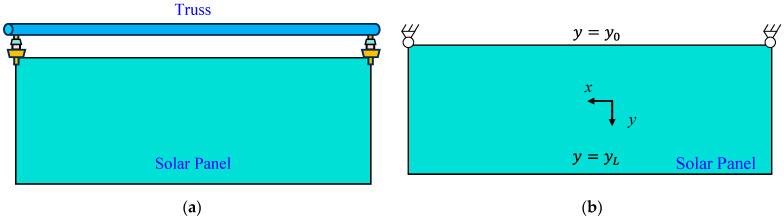
The boundary condition of panel components: (**a**) the connection between the truss and panel; (**b**) the isolated panel in ITSs.

In the case of the space truss structure, three primary beams along the *x* direction can be regarded as cantilever beams, in which the deflection and slope at fixed left ends are nominal zero, i.e., $w_{0}={tan\theta }_{x_{0}}=0$. Hence, the deformation of the truss can be reconstructed based on Equation (6). For panel components in ITSs, as shown in Figure 3, boundary conditions deviate from classic scenarios. The panel is clamped to the edge of the space truss at only two attachment points. Deflection curves of the “two-node fixed” configuration are unconstrained within the range $\left(y_{0},\;\;y_{L}\right]$. Consequently, initial values required for Equation (6) cannot be determined directly.

**Figure 4:**
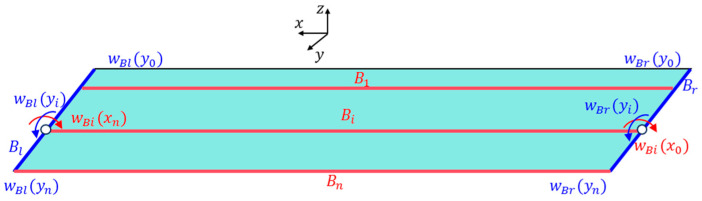
Deformation reconstruction for panels with complex boundary conditions.

To tackle this issue, this study proposes a deformation reconstruction strategy tailored for panels with complex boundary conditions in ITSs. As illustrated in Figure 4, the panel can be regarded as a cluster of space broken-line beams, where left and right edges of the panel are designated as $B_{l}$ and $B_{r}$, respectively. Beams along the long axis of the panel are denoted as $B_{i}$. Due to different axial directions of beams $B_{i}$ and $B_{l}$, normal displacements of panel components are represented as $w_{Bi}\left(x\right)$ and $w_{Bl}\left(y\right)$, respectively. In the case of $B_{l}$ and $B_{r}$, their deformation can be treated as typical cantilever beams with angles at fixed starting points set to zero. Hence, deflection curves of $B_{l}$ and $B_{r}$ can be determined based on the discrete strain information provided that initial deflections $w_{Bl}(y_{0})$ and $w_{Br}(y_{0})$ are known. Additionally, the deformation at the interface between the truss and the plats is assumed to be negligible, leading to initial deflections $w_{Bl}(y_{0})$ and $w_{Br}(y_{0})$ being equal to deflection values at the corresponding connection points on the space truss. Moreover, at connections between $B_{i}$ beams and $B_{l}$ or $B_{r}$ beams, the deflection is continuous, whereas angles are discontinuous. Mathematically, this relationship is expressed as

(7) $$\begin{array}{c} w_{Bl}\left(y_{i}\right)=w_{Bi}\left(x_{n}\right)\\ w_{Br}\left(y_{i}\right)=w_{Bi}\left(x_{0}\right)\\ \theta_{Br}\left(y_{i}\right)\neq \theta_{Bi}\left(x_{0}\right) \end{array}$$

For an arbitrary $B_{i}$, based on Equation (6), it is noted that

(8) $$\frac{\partial w\left(x\right)}{\partial {tan\theta }_{x_{i}}}=(x_{i+1}-x_{i})$$

**Figure 5:**
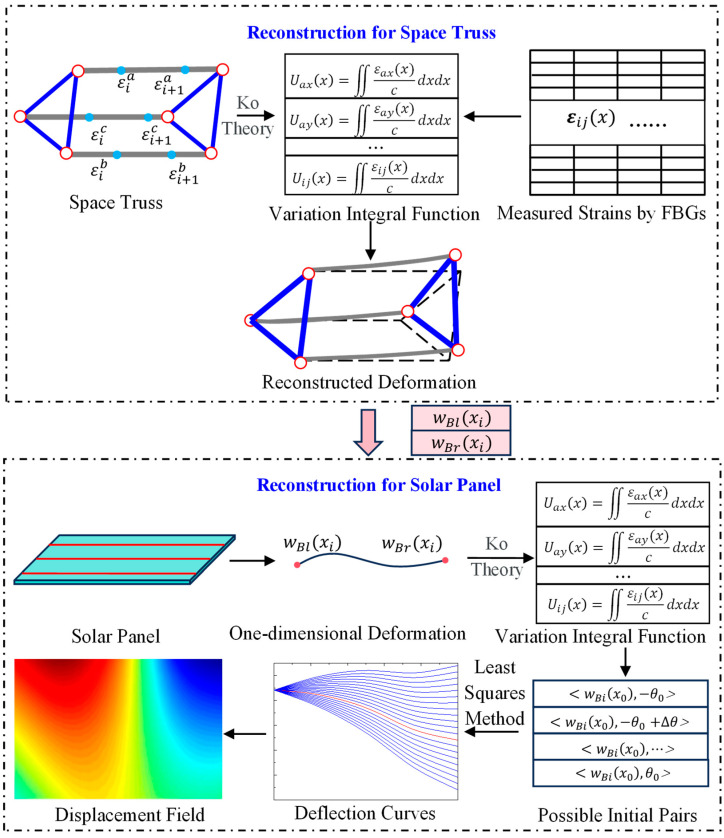
The flowchart of the proposed deformation reconstruction strategy.

For a given sensor configuration, ($x_{i+1}-x_{i}$) is a constant value for each integral range. Moreover, in the case of the small deformation assumption, the function tan⁡(θ) is monotonic. Therefore, it can be concluded that the deflection function of the $B_{i}$ beam is monotonic with respect to the unknown item ${tan\theta }_{x_{i}}$. Using this property, various initial values pairs <$w_{Bi}\left(x_{0}\right)$, $\theta_{i}$> can be obtained, each generating a distinct deflection curve. For each curve, the displacement at the end $w_{Bi}^{\prime }\left(x_{n}\right)$ is different. The least square method is carried out to compare errors between calculated $w_{Bi}^{\prime }\left(x_{n}\right)$ and target $w_{Bi}\left(x_{n}\right)$. The optimal initial value pair is determined by minimizing the error, allowing for the reconstruction of ideal deflection curves. Following this strategy, normal displacements of any three different deflection curves of the panel with complex boundary conditions can be reconstructed. The torsion of the panel is calculated as

(9) $$\varphi \left(x_{i}\right)={sin}^{-1}(\frac{y_{n}-y_{1}}{d})$$

where $\varphi \left(x_{i}\right)$ represents the torsion at $x_{i}$. $y_{n}$ and $y_{1}$ are the identified deflection curves of the leading and trailing edges, respectively. d denotes the width of the panel. In addition, the displacement field within the panel can be represented using linear interpolation and extrapolation algorithms to ensure a smooth and continuous deformation profile. The schematic representation of the proposed deformation reconstruction strategy for ITSs is shown in Figure 5.

## 3. Numerical Scenarios

### 3.1. Numerical Model

**Figure 6:**
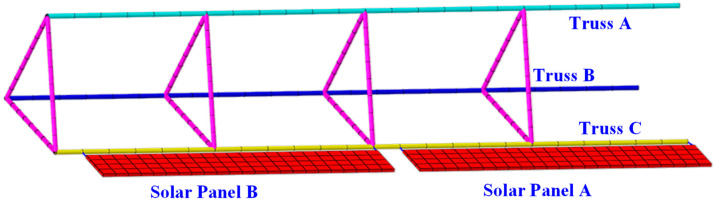
The numerical model of ITSs.

As illustrated in Figure 6, the structure comprises two components: the primary space truss framework and attached solar panels. Additional elements, such as CubeSats and antenna systems, are modeled as concentrated forces applied to the truss structure for simplification. The elementary unit cell of the periodic truss adopts a triangular prism configuration, which is characterized by a cross-sectional equilateral triangle with side lengths of 1000 mm. The structural unit cell extends 1250 mm along the *x*-axis. ITSs consist of four truss cells, yielding a total length of 5 m. Two plates are affixed to Truss C. Each plate has dimensions of 2300 mm in length, 350 mm in width, and 30 mm in thickness. The first plate is attached to the first two spans, while the second plate is connected to the last two spans. To facilitate folding and deployment, two plates are designed to operate independently of one another.

**Figure 7:**
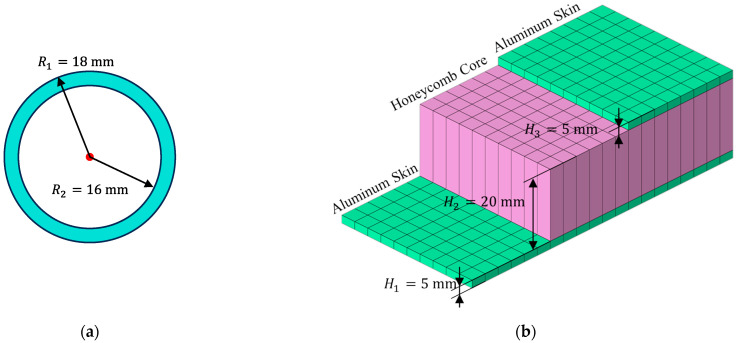
Cross-sections of main components in ITSs: (**a**) the cross-section of space trusses; (**b**) the stack of sandwich panels.

Space truss structures are fabricated from carbon-based materials with detailed material properties listed in Table 1. All beam elements in the numerical model share a consistent cross-sectional geometry where the specific profile is tubular and depicted in Figure 7. It is noted that the coefficients of thermal expansion of the three trusses are distinct. Additionally, solar panels are modeled as honeycomb sandwich plates with a honeycomb core thickness of 20 mm and aluminum skin thickness of 5 mm. Considering the thermal–mechanical load in this analysis, the reference temperature for all materials is set to 20 °C.

In the numerical model, the truss structure is discretized using beam elements, while solar panels are meshed with CQUAD4 elements incorporating layered composite properties. Connections between the truss and panels are modeled using multiple-point constraints (MPCs) implemented through RBE2 elements, which fully constrain both translational and rotational degrees of freedom at connection points. Based on results of convergence analysis, characteristic mesh sizes for beams and composite elements are set to 210 mm and 100 mm, respectively.

### 3.2. Load Scenarios

To comprehensively validate the proposed deformation reconstruction strategy, various load scenarios applied to ITSs are investigated, including multi-point and uniformly distributed mechanical loads. Additionally, scenarios involving a combined thermal–mechanical load are also analyzed.

**Figure 8:**
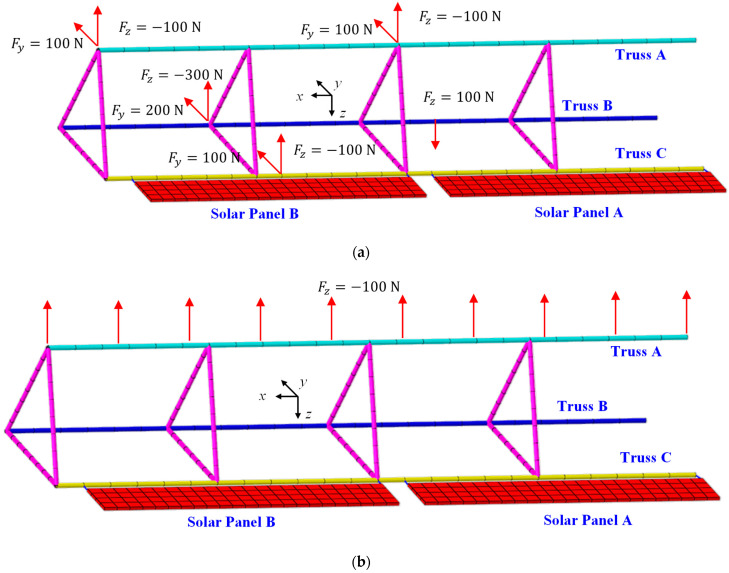
Mechanical load scenarios: (**a**) multiple point loads; (**b**) uniformed loads.

**Figure 9:**
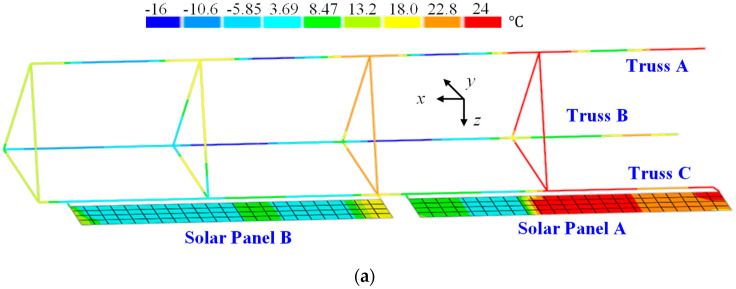

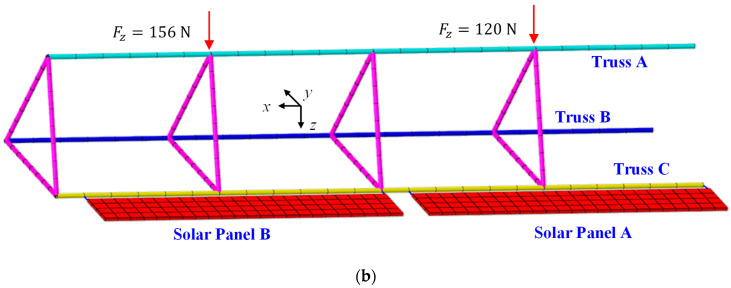
The thermal–mechanical load scenario: (**a**) the temperature field of ITSs; (**b**) internal forces induced by other components.

In the case of the first scenario, load cases are plotted in Figure 8. For the second scenario, the analysis incorporates thermal loads combined with multi-point mechanical loads, as depicted in Figure 9. The temperature field depicted in Figure 9a represents a typical scenario corresponding to the maximum temperature gradient experienced by ITSs. Additionally, other components undergo deformation influenced by this thermal field. The extent of deformation varies among components due to differences in their geometry and material properties. To ensure displacement continuity across different components of ITSs, internal forces arise at attachment points. These internal forces, which influence the truss structure, are represented in the model as multiple concentrated forces, as shown in Figure 9b.

For both scenarios, boundary conditions are consistent. The left ends of the three trusses are fixed at *x* = 0, while each panel is attached to an edge of the truss at two joint points, which is modeled using MPCs. For space truss structures, strains are measured at each node of beam elements, and strains at leading and trailing edges of solar panels are extracted to reconstruct the deformation.

## 4. Results and Discussion

In this section, various results are presented and analyzed to validate the accuracy of the proposed deformation reconstruction strategy. The error based on root mean square (RMS) is employed as a metric to quantify the difference between the reconstructed deformation w(x) and the nominal displacement $w^{\prime }(x)$, which is defined as

(10) $$Error(RMS)=\sqrt{\frac{\sum_{i=1}^{n}{\left(w\left(x_{i}\right)-w^{\prime }(x_{i})\right)}^{2}}{n}}$$

### 4.1. Reconstruction Results in Mechanical Loads

**Figure 10:**
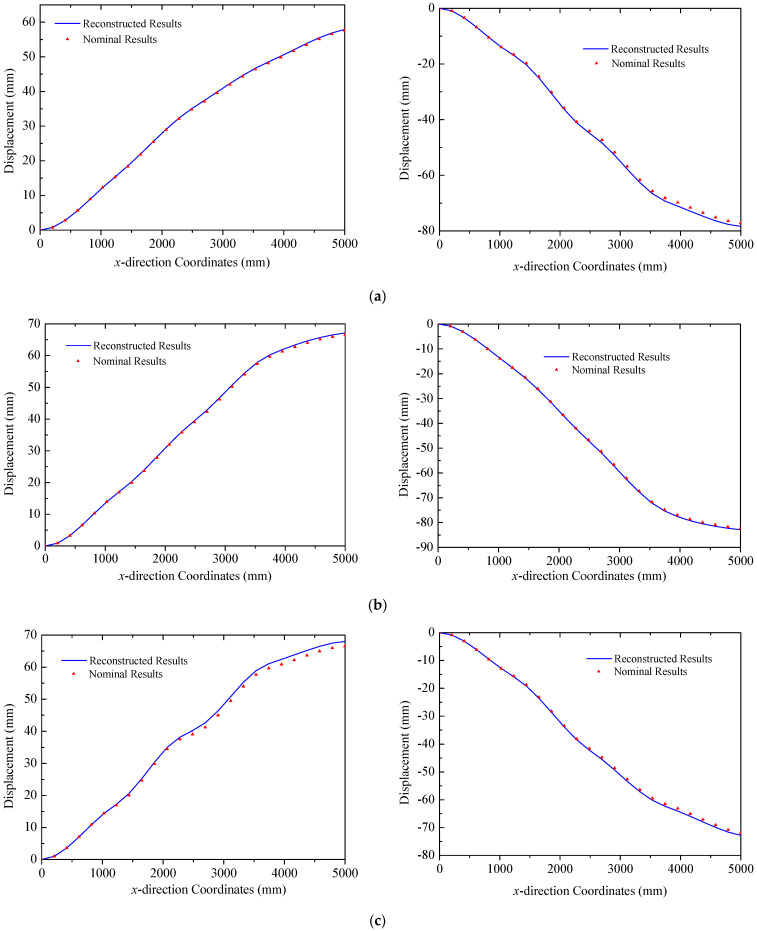
Reconstruction results of truss structures (multi-point loads): (**a**) Truss A: left *y* direction. right *z* direction; (**b**) Truss B: left *y* direction. right *z* direction; (**c**) Truss C: left *y* direction. right *z* direction.

In the case of the multi-point load scenario, loads are applied at three trusses in both the *y* and *z* directions. The reconstructed deformations obtained by the proposed strategy of three trusses are plotted in Figure 10, in which nominal results are calculated by the commercial finite element software. Deformation modes of three trusses exhibit consistency with upward bending along the negative *z* direction and rightward bending along the positive *y* direction. Comparisons of reconstructed and nominal results demonstrate that the proposed strategy has the ability to effectively reconstruct the deformation of triangular truss structures in ITSs under multi-point loads. For Truss A, the errors (RMS) defined in Equation (10) are 0.22 mm and 0.85 mm in the two directions, respectively. The corresponding errors (RMS) for Truss B are 0.39 mm and 0.38 mm. In the case of Truss C mounted with solar panels, indicators are 1.06 mm and 0.59 mm, respectively. The maximum error in this case occurs at x = 3950  mm, in Truss C, with an error of only 1.61 mm. Additionally, due to the influence of concentrated loads, deflection curves of trusses exhibit abrupt changes at the locations of applied loads, which is captured in both reconstructed and nominal results. For instance, in the deflection of Truss A along the *z* direction, an abrupt change is observed at x = 2500  mm, corresponding to the location of a concentrated load in Figure 8a.

**Figure 11:**
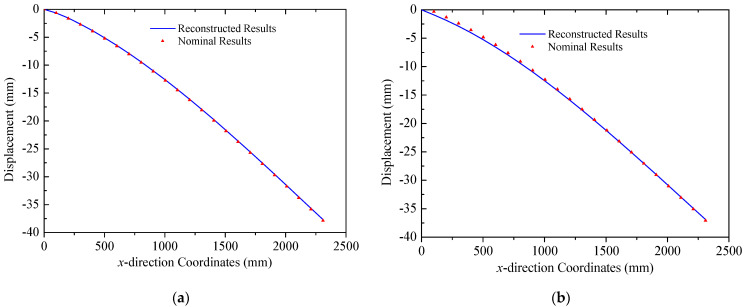
Reconstruction results of solar panel A (multi-point loads): (**a**) leading edge *y* = 0 mm; (**b**) trailing edge *y* = 350 mm.

**Figure 12:**
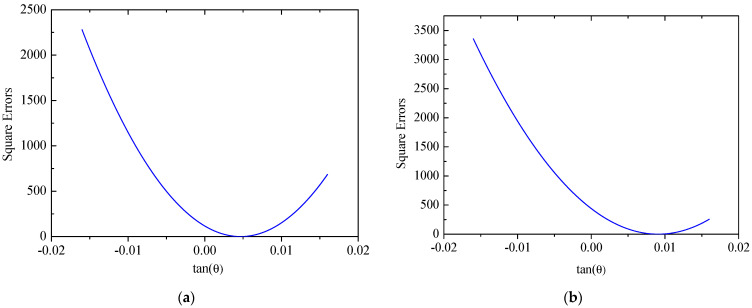
Errors of the solar panel A: (**a**) leading edge *y* = 0 mm; (**b**) trailing edge *y* = 350 mm.

**Figure 13:**
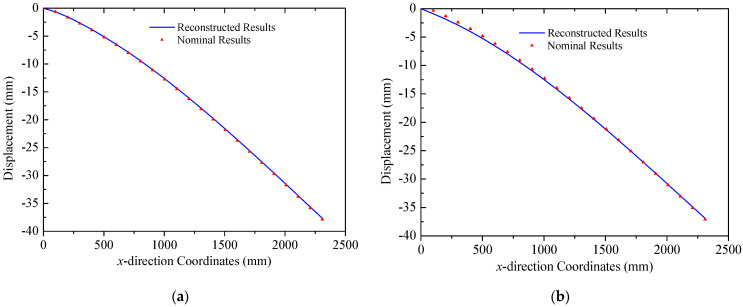
Reconstruction results of the solar panel B (multi-point loads): (**a**) leading edge *y* = 0 mm; (**b**) trailing edge *y* = 350 mm.

**Figure 14:**
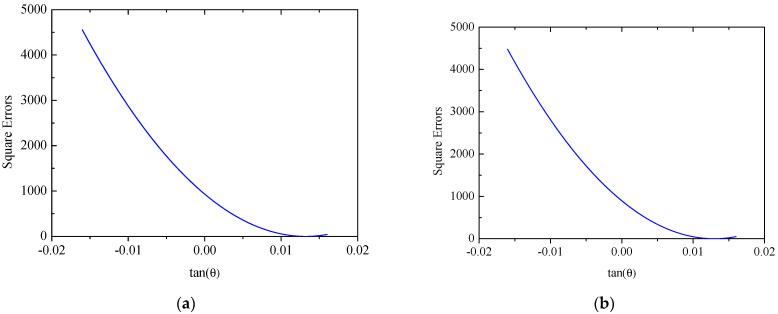
Errors of the solar panel B: (**a**) leading edge *y* = 0 mm; (**b**) trailing edge *y* = 350 mm.

**Figure 15:**
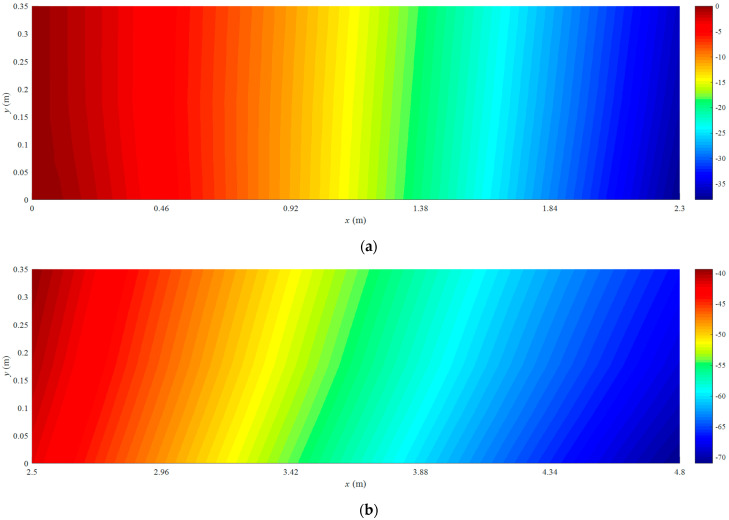
Reconstructed displacement fields: (**a**) solar panel A; (**b**) solar panel B.

The coordinates of two mounting points of solar panel A on Truss C are x = 0 mm and x = 2300  mm with their respective displacements denoted as $w_{1}$ = 0 mm and $w_{2}$ = 38.45 mm. Based on the small deformation hypothesis, the optimization interval for initial values tan⁡(θ) is set to [−0.16, 0.16]. Using the least squares optimization method shown in Figure 4, normal displacements at leading and trailing edges of the panel are obtained, which are illustrated in Figure 11. The errors (RMS) of the reconstructed results are 0.12 mm and 0.32 mm for the leading and trailing edges, respectively. Consequently, it can be concluded that the proposed strategy can accurately reconstruct the displacement field of panel structures with two-point fixed boundary conditions. Moreover, Figure 12 illustrates errors between reconstructed and nominal responses at the endpoint of the deflection curve as a function of tan⁡(θ) during the least squares optimization process. For both leading and trailing edge cases, a unique minimum point is observed, aligning with Equation (8). For solar panel A, the optimal values of tan⁡(θ) are 0.0047 and 0.0091 for leading and trailing edges, respectively. A similar trend is observed for solar panel B, which is shown in Figure 13. Errors (RMS) for this panel are 0.23 mm and 0.58 mm, respectively with the maximum error of 0.73 mm occurring at x = 2500  mm on the leading edge of plate B. These results indicate that the proposed method effectively reconstructs the displacement field of flat plates at intermediate positions. The optimization process for tan⁡(θ) is depicted in Figure 14, confirming the existence of unique optimal solutions. Based on the reconstructed deflections of panels A and B, the entire displacement field can be obtained by using linear interpolation, and the fields are illustrated in Figure 15.

**Figure 16:**
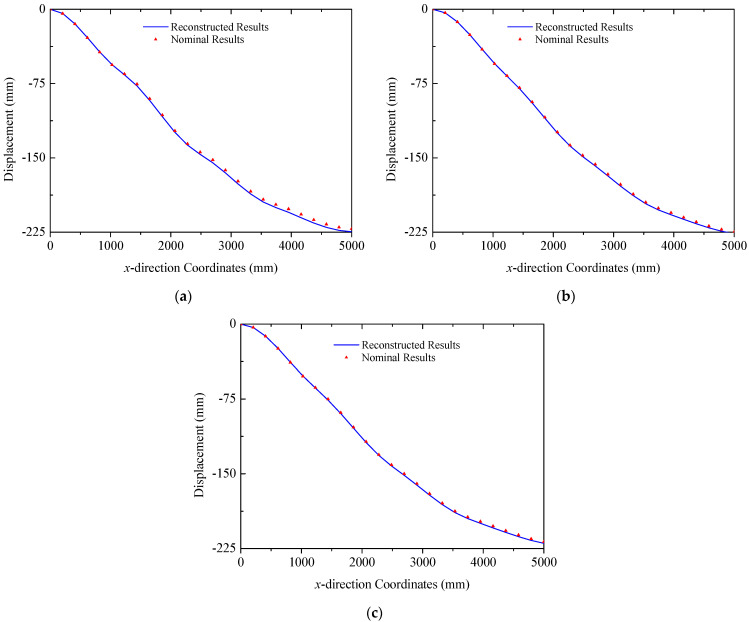
Reconstruction results of truss structures (uniformed loads): (**a**) Truss A; (**b**) Truss B; (**c**) Truss C.

**Figure 17:**
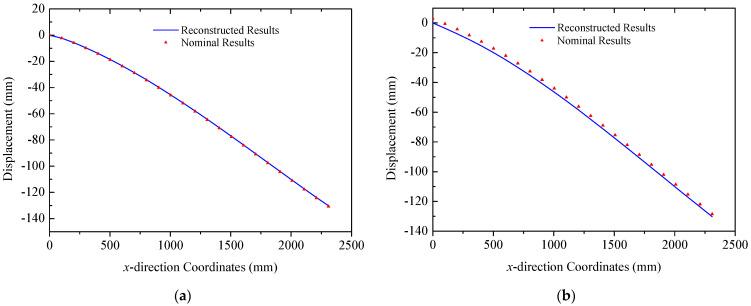
Reconstruction results of the solar panel A (uniformed loads): (**a**) the leading edge *y* = 0 mm; (**b**) the trailing edge *y* = 350 mm.

**Figure 18:**
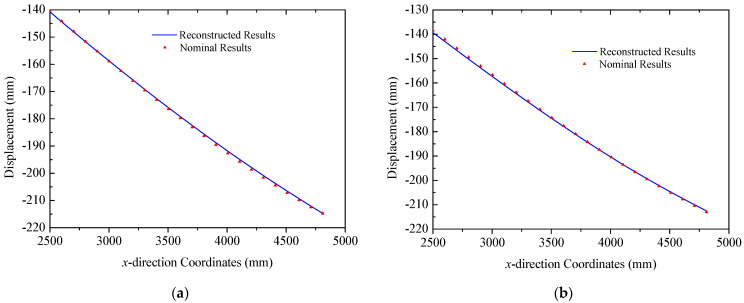
Reconstruction results of the solar panel B (uniformed loads): (**a**) the leading edge *y* = 0 mm; (**b**) the trailing edge *y* = 350 mm.

In the case of the uniform load in Figure 8b, primary deformations of triangular trusses are bending along the *z* direction, while the deformation in the *y* direction is negligible, since the amplitude is far less than that of the *y* direction. The deformation reconstruction results for three trusses are presented in Figure 16. Because of the higher magnitude of the uniform load compared to the multi-point load in Figure 8a, the peak amplitude of the response increases to approximately 225 mm. A comparison in Figure 16 also demonstrates the effectiveness of the reconstruction algorithm. In this case, the errors (RMS) for three trusses are 2.28 mm, 1.33 mm, and 1.22 mm, respectively, in which the maximum error is 3.47 mm in Truss A. Reconstructed and nominal displacements of panels A and B under the uniform load are shown in Figure 17 and Figure 18, respectively. Their errors (RMS) are 0.29 mm, 2.48 mm, 0.51 mm, and 0.48 mm for four different results. The maximum error for panel structures in this case does not exceed 3.10 mm, which further demonstrates the accuracy of the proposed reconstruction strategy.

### 4.2. Reconstruction Results in the Thermal–Mechanical Load

**Figure 19:**
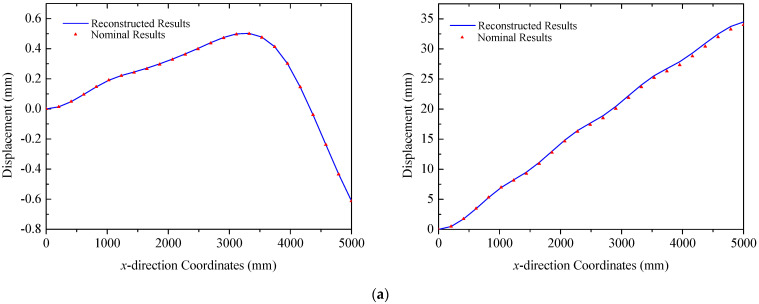

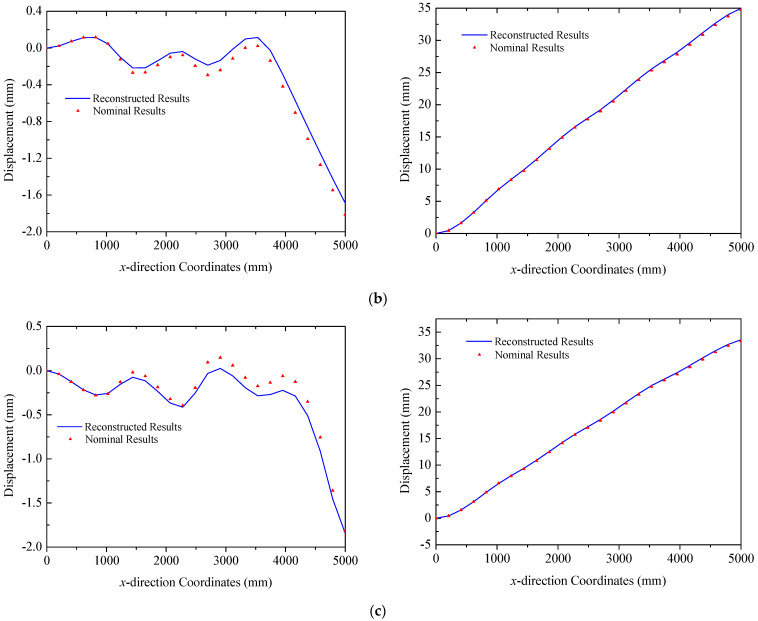
Reconstruction results of truss structures (thermal–mechanical loads): (**a**) Truss A: left *y* direction. right *z* direction; (**b**) Truss B: left *y* direction. right *z* direction; (**c**) Truss C: left *y* direction. right *z* direction.

In this section, the proposed strategy is adopted to reconstruct the deformation of ITSs under combined thermal–mechanical loads, where the maximum temperature gradient in the field reaches approximately 40 °C. The deformation modes in this scenario are more complicated compared to those induced by pure mechanical loads, which is particularly evident in the displacement in the *y* direction. As shown in Figure 19, the primary deformation of the truss structure is dominated by the *z* directional component. For Truss A, the values of error (RMS) are 0.0021 mm and 0.32 mm with a maximum error of 0.53 mm. In the case of Truss B, indicators are 0.085 mm and 0.18 mm, respectively. For Truss C, errors (RMS) are less than 0.17 mm for the two figures in Figure 19c. These comparisons validate the capability of the proposed method in reconstructing deformations of triangular trusses under thermal–mechanical loads.

**Figure 20:**
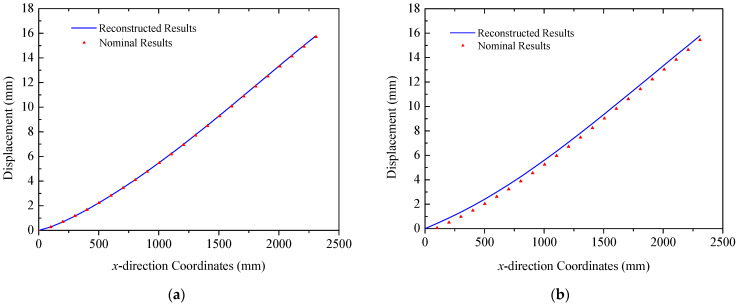
Reconstruction results of the solar panel A (thermal–mechanical loads): (**a**) the leading edge *y* = 0 mm; (**b**) the trailing edge *y* = 350 mm.

**Figure 21:**
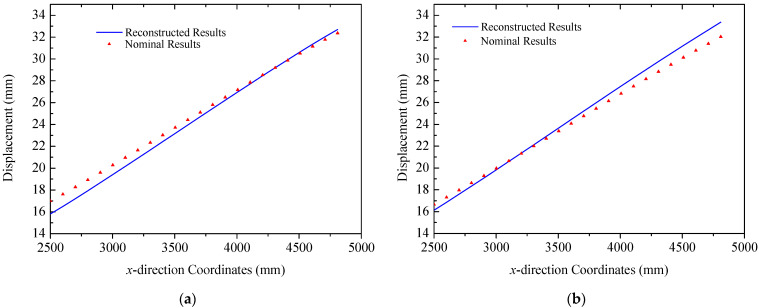
Reconstruction results of the solar panel B (thermal–mechanical loads): (**a**) the leading edge *y* = 0 mm; (**b**) the trailing edge *y* = 350 mm.

Additionally, it is noted that stress and strain distributions in Truss B and Truss C are complex since they are under thermal loads. Linear interpolation of the strain component between two sensors may induce errors, which prevents the proposed algorithm from fully capturing the intricate deformation modes of the structure. To solve this issue, the accuracy of the reconstruction strategy could be enhanced by either reducing the distance between sensors or adopting a higher-order interpolation to establish the function of the strain distribution. Moreover, as indicated by the temperature field in Figure 9a, the temperature gradient in Truss C is greater than that in the other two trusses. Nevertheless, the asymmetric design of the structural material properties mitigates the incongruity among three trusses, resulting in displacement in both the *y* and *z* directions comparable to those of the others. The design can effectively minimize the torsional deformation of triangular truss structures. For the two solar panels in ITSs, the reconstructed normal displacements of the trailing and leading edges are depicted in Figure 20 and Figure 21 for panel A and panel B, respectively. The comparison of reconstructed and nominal results validates the effectiveness of the proposed method for panels in the case of coupling loads, where the maximum error is less than 1.34 mm.

## 5. Conclusions

This study presents a deformation reconstruction strategy for integrated truss structures (ITSs) by integrating Ko displacement theory with the least squares optimization. The methodology is applied to an assembly consisting of a primary truss structure and solar panels with two-point fixed boundary conditions. To validate the proposed approach, comparisons between nominal and reconstructed displacements are conducted under three load scenarios: multi-point loads, uniform loads, and thermal–mechanical loads. The main conclusions are summarized as follows:

(1) The proposed strategy effectively reconstructs ITSs deformations under complex load conditions. For the primary truss structure, the method accurately captures bending deformations of three trusses in both principal directions across all studied load scenarios. The maximum error based on the root mean square is limited to 2.28 mm, with a displacement of 225 mm, demonstrating high reconstruction accuracy. For flat panel structures mounted to trusses with two-point fixed boundaries, the least square optimization successfully solves the integral equation for deflection curves even with unknown initial values. This approach enables accurate one-dimensional deflection curve reconstruction, which serves as a basis for generating displacement field contour plots. The maximum error for panel reconstruction is only 3.10 mm, highlighting the potential of the proposed strategy for in-orbit deformation monitoring and reconstruction.
(2) Under thermal–mechanical loads, structural stress and strain distributions become more intricate compared to purely mechanical loads, resulting in an oscillatory bending deformation mode for trusses. To enhance the accuracy of the deformation reconstruction in such scenarios, reducing sensor spacing or employing higher-order interpolation functions to model strain distributions more accurately may be necessary.

The results highlight the applicability of the proposed method for ITSs, providing a foundation for reliable in-orbit performance monitoring and deformation identification. Future work may explore the further optimization of sensor configurations and algorithms to address challenges posed by highly nonlinear deformation behavior under complex load conditions.

## Figures and Tables

**Table 1:** Material properties of main components in ITSs.

Material of Truss Structures		
E=90,000 Mpa	G=34,615 Mpa	ν=0.3
$\alpha_{TrussA}=$3×10^−7^/℃	$\alpha_{TrussB}=$3×10^−7^/℃	$\alpha_{TrussC}=$3.5×10^−6^/℃
Material of solar panels		
Aluminum Skin		
E=70,000 Mpa	ν=0.32	$\alpha_{Skin}=2.38\;\times$10^−5^/℃
Honeycomb Core		
$E_{1}=$0.001 Mpa	$E_{2}=$0.001 Mpa	$\nu_{12}\;$ = 0.3
$G_{12}$ = 0.001 Mpa	$G_{1z\;}$ = 111 Mpa	$G_{2z}$ = 111 Mpa
	$\alpha_{1}$ = 2.38 ×10^−5^/℃	$\alpha_{2}$ = 2.38 ×10^−5^/℃

## Data Availability

Data will be made available on request.

## Abbreviations

The following abbreviations are used in this manuscript:

ITS	integrated truss structures
FBG	Fiber Bragg Grating
DOF	degree of freedom
iFEM	inverse finite element method
BP	back propagation
MPC	multiple-point constraints
RMS	root mean square
